# Color radiography in lung nodule detection and characterization: comparison with conventional gray scale radiography

**DOI:** 10.1186/s12880-016-0155-7

**Published:** 2016-08-22

**Authors:** Inyoung Song, Jeong Geun Yi, Jeong Hee Park, Kyung Soo Lee, Myung Jin Chung

**Affiliations:** 1 Department of Radiology, Konkuk University School of Medicine, Seoul, 143-729 South Korea; 2 Department of Radiology, Samsung Medical Center, Sungkyunkwan University School of Medicine, 81 Irwon-ro, Gangnam-gu, Seoul, 135-710 South Korea; 3 Department of Digital Health, SAIHST, Sungkyunkwan University, Seoul, 135-710 South Korea

**Keywords:** Radiography, Color radiography, Dual-energy subtraction radiography

## Abstract

**Background:**

To compare the capability of lung nodule detection and characterization between dual-energy radiography with color-representation (DCR) and conventional gray scale chest radiography (GSR).

**Methods:**

A total of 130 paired chest radiographs (DCR and GSR) obtained from 65 patients (14 with normal scans and 51 with pulmonary nodules) were evaluated. After analysis, 45 non-calcified and 21 calcified nodules were identified. DCR was obtained by adding color space within material-decomposed data (blue for high attenuation and red for low attenuation) and by compounding the manipulated data to one color image. Three radiologists marked suggested nodules on radiographic images and assessed the level of confidence of lesion presence and probability of nodule calcification by using a nine-point rating scale. The jackknife active free-response receiver operating characteristics (JAFROC) analysis was used to evaluate lesion detectability, and multi-reader multi-case receiver operating characteristics (MRMC ROC) analysis was used for the evaluation of the accuracy of nodule calcification prediction.

**Results:**

Figures of merit (FOM) from JAFROC was 0.807 for DCR and 0.811 for GSR, respectively; nodule detectability was not significantly different between DCR and GSR (*p* = 0.93). Areas under curve (AUC) from MRMC ROC were 0.944 for DCR and 0.828 for GSR, respectively; performance of DCR in predicting lung nodule calcification was significantly higher than that of GSR (*p* = 0.04).

**Conclusions:**

DCR showed similar performance in terms of lung nodule detection compared with GSR. However, DCR does provide a significant benefit in predicting the presence of nodule calcification.

## Background

Conventional radiography (CR) is still the most commonly used imaging tool for detection and evaluation of pulmonary nodules owing to its wide availability, low cost, and low radiation dose in spite of its inferior performance compared to computed tomography (CT) [1, 2]. The detection and characterization of pulmonary nodules are often difficult against the background of overlapping clutters, such as ribs and other bones, mediastinum or pulmonary vessels [2–5]. Dual-energy radiography (DER) is one of the proposed methods for improving lung nodule conspicuity using two different X-ray spectra, making it possible to separate an image into soft-tissue image and bone image (e.g. material decomposition) [6–12].

If the information about material density of pulmonary nodule is expressed by different color on DER, the conspicuity of nodule would be increased. Thus, we hypothesized that: (1) DER with color-representation (DCR), which can simultaneously provide both anatomic and density information, might provide added value to characterize pulmonary nodule and (2) DCR might hamper nodule detectability due to misregistration artifacts from dual exposure technique when compared with CR. Therefore, the aim of this study was to compare the capability of lung nodule detection and characterization between DCR and conventional gray scale chest radiography (GSR).

## Methods

Our institutional (Samsung Medical Center [SMC]) review board approved our study (SMC 2015-11-005) with a waiver of informed consent.

### Subjects

We reviewed images from 272 patients (age > 18 years) who underwent chest CT and DER studies for the evaluation of known or suspected pulmonary nodules between Mar 2005 and Dec 2007. Patients were excluded if there were any abnormal findings suspicious for 1) active inflammation/infection (consolidation, clustered centrilobular nodules, ground-glass opacity, pleural effusion), 2) post-infectious scar or stable tuberculosis (fibrotic bands, traction bronchiectasis, architectural distortion), 3) pulmonary fibrosis (subpleural honeycombing, reticulation), 4) any other structural distortion (severe bronchiectasis, lobar atelectasis, segmentectomy/lobectomy state) on CT scan. Patients with pulmonary nodule that measured < 4 mm or > 40 mm on CT were also excluded. As a result, 65 patients (14 patients with normal scans and 51 patients with pulmonary nodules) were included.

### DER and CT imaging

DER was performed using a flat panel detector system (Definium 8000, GE Healthcare, Waukesha, WI). The detector has an image size of 41 × 41 cm with 200 mm pixels. DER were acquired with respiration suspended in deep inspiration using the standard dual shot method (120 kVp and 60 kVp) with a 200-millisecond delay between the high- and low-energy exposures. The dual-energy examination consists of the standard digital posteroanterior gray scale radiograph (GSR) which is the equivalent of an 80-kVp examination as well as the soft tissue image (bone subtracted) and the bone image (soft tissue subtracted). The entrance surface doses for the DER were recorded by dose area product meter and the average of dose was 0.11 ± 0.04 mGy. Various CT scanners were used; 40-detector scanner (Brilliance-40, Philips Medical System, Eindhoven, The Netherlands), 16-detector scanners (LightSpeed 16; LightSpeed QX/I, both GE Healthcare, Waukesha, WI, USA), and an eight-detector scanner (LightSpeed Ultra, GE Healthcare). Scanning was performed from the thoracic inlet to the middle portion of the kidneys in supine position. Images were reconstructed with a high-spatial-frequency algorithm. CT image parameters were as follows: detector collimation, 1.25 or 0.625 mm; field of view, 36 cm; beam pitch, 1.35 or 1.375; gantry speed, 0.5 or 0.6 s/rotation; 120 kVp; 150–200 mA; and reconstruction interval, 1–2.5 mm. Image reformation with a 2.5-mm section thickness for transverse images and a 2.0-mm section thickness for coronal images was performed.

### Image postprocessing

Our proposed DCR visualization method is based on the overlay of color on original DER image. The DER unit produces a material-decomposed data. A color space, derived from compounding existing manipulated data, was added on material-decomposed data according to the attenuation value (blue for high attenuation and red for low attenuation). The color image was then overlaid to unprocessed gray scale DER.

### Observer performance study

Three independent, board-certified radiologists with 3, 5, and 6 years of experience in chest imaging, respectively, evaluated the radiographs for the presence of pulmonary nodules using the free-response paradigm [13, 14]. A total of 130 images (65 pairs of GSR and DCR from each patient) were reviewed. Images were blinded and randomly sorted, and divided into two subgroups. Each subgroup included one random image within a pair and vice versa. Each observer reviewed subgroup 1 or 2 and vice versa with 1 month interval. The observers were informed about possible presence of multiple nodules per case. Images were evaluated using a standard picture archiving and communication system (Centricity RA 1000; GE Healthcare, Barrington, IL, USA). The observers were able to adjust the window level and the window width at their discretion. They were asked to identify (mark) suspected nodules on the radiographs and to grade the confidence of presence for each marked nodule on a scale from score one to nine, where score nine represented the highest degree of confidence (definitely a nodule) and score one represented the lowest degree of confidence (probably not a nodule). They also graded the confidence of nodule calcification for each marked nodule using scale from score one to nine, where score nine represented the highest degree of confidence (definitely calcified nodule) and score one represented the lowest degree of confidence (probably not calcified nodule).

### The reference standard

Before the completion of the detection study, the reference standard was created in consensus by two experienced thoracic radiologists (M. J. C. and I. S.), who had 12 and 4 years of chest CT interpretation experience, respectively. First, chest CT examinations were used for nodule detection. The nodules were identified using axial and coronal reformations in all cases. The size, number and calcification of pulmonary nodules were assessed. A nodule was considered calcified if calcification was visually detected in mediastinal window settings (level + 40, width 400) [15]. Based on the nodule localization on the multidetector CT images, the nodules were marked on the radiographs to verify the true locations. Some of the nodules could not be seen but were marked at the locations where they should have been according to the multidetector CT images.

### Statistical analysis

All marks made by the readers on radiographs were compared with findings on chest CT as the reference standard. The jackknife alternative free-response receiver operating characteristic (JAFROC) method was used to evaluate nodule detectability on GSR and DCR (JAFROC freeware, version 4.1, Dev Chakraborty). This method includes the calculation of a figure of merit (FOM) averaged across all readers for each investigated modality and computes the 95 % confidence interval for the intermodality differences of the FOMs. The exact *p* value is calculated using F statistics.

The accuracy of prediction of nodule calcification was determined by multi-reader, multi-case receiver operating characteristic (MRMCROC) methodology [16, 17]. Dorfman-Berbaum-Metz (DBM) MRMC beta 2.3 (20) was used for calculating the areas under curve (AUC) value.

## Results

**Fig 1:**
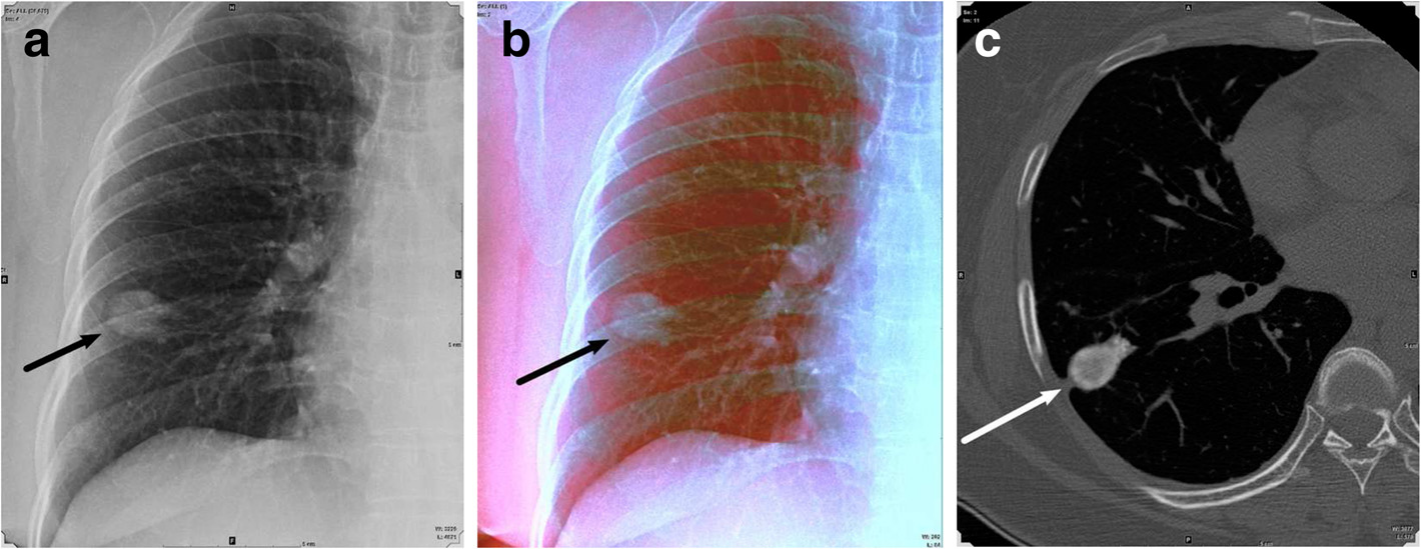
Conventional gray scale radiography (GSR) and dual-energy radiography with color-representation (DCR) of a 60-year-old woman. **a** Conventional GSR image shows a nodular opacity in right lower lung zone (*arrow*). **b** DCR image shows distinct blue color of the nodular opacity (*arrow*) indicating calcified nodule. **c** Axial CT image shows a calcified granuloma in the right lower lobe (*arrow*)

**Fig 2:**
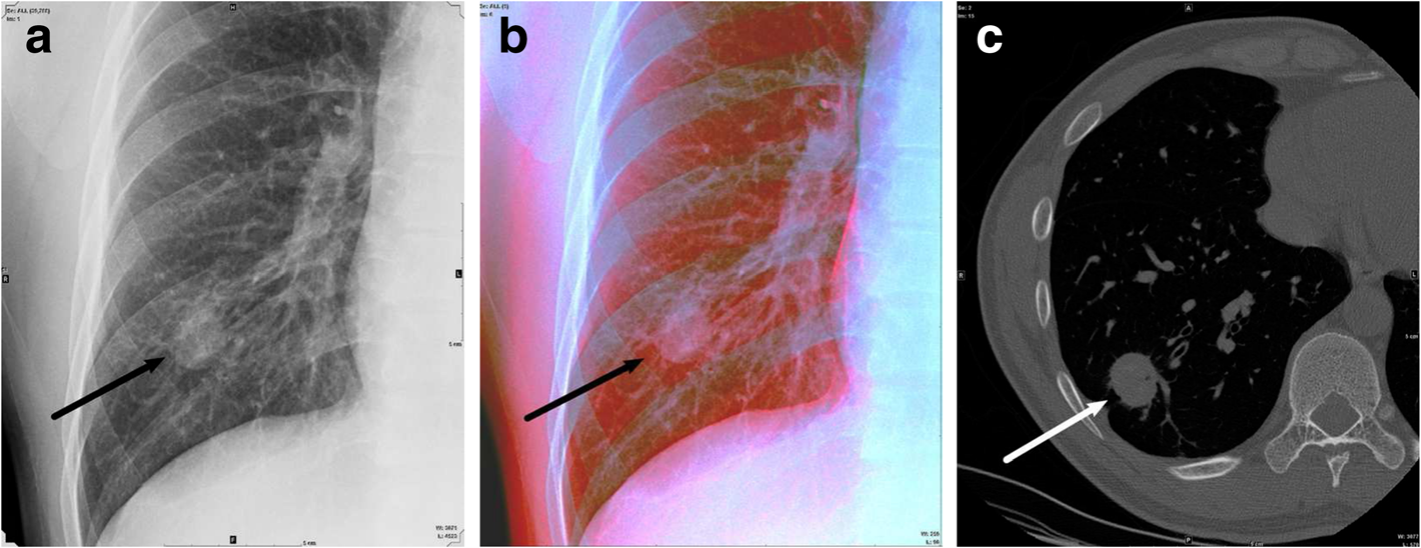
Conventional gray scale radiography (GSR) and dual-energy radiography with color-representation (DCR) of a 34-year-old man. **a** Conventional GSR image shows a nodular opacity in right lower lung zone (*arrow*). **b** DCR image shows distinct pink color of the nodular opacity (*arrow*) indicating non-calcified nodule. **c** Axial CT image shows a non-calcified nodule in the right lower lobe (*arrow*). This nodule was pathologically proved to be a sclerosing hemangioma

A total of 66 nodules (45 non-calcified and 21 calcified nodules) were identified in 51 patients. All were solid nodules, with number of nodules per patient ranging from one to four (Figs. 1 and 2).

### Nodule detection

**Fig 3:**
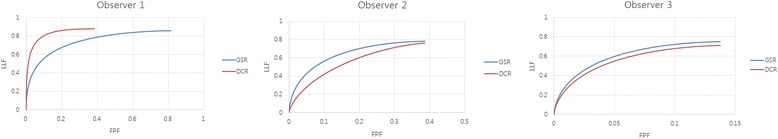
Average free-response ROC curves for nodule detectability on dual-energy radiography with color-representation (DCR) and conventional *gray* scale radiography (GSR) images by each observer. *FPF* false positive fraction, *LLF* lesion localization fraction

The observers’ performance for the nodule detection in DCR and GSR images was shown by the average free-response ROC curves in Fig. 3. The reader-averaged values of the JAFROC FOM for the detection of pulmonary nodules were not significantly different between DCR and GSR (*p* = 0.93). When GSR images were given to the readers, the reader-averaged FOM was 0.811 while the reader-averaged FOM was 0.807 when DCR images were added. FOM values for both interpretation sessions and radiologists are shown in Table 1.

**Table 1:** Figure-of-merit values obtained from the JAFROC analysis for three radiologists in detecting lung nodules on conventional gray scale radiography (GSR) and dual-energy radiography with color-representation (DCR)

Observer	Gray scale radiography	Dual-energy radiography with color-representation
1	0.811	0.869
2	0.800	0.757
3	0.821	0.794
Average	0.811	0.807

The difference in figure-of-merit values between GSR and DCR imaging was not statistically significant (*p* = 0.928)

*JAFROC* jackknife alternative free-response receiver operating characteristic

### Nodule characterization

The MRMCROC analysis for the accuracy of prediction of nodule calcification revealed that the DCR improved the AUC values for all readers. Average AUCs were 0.828 with GSR and 0.944 with DCR. The differences were statistically significant between GSR and DCR (*p* < 0.044). AUC values for both interpretation sessions and radiologists are shown in Table 2.

**Table 2:** Area under the receiver operating characteristic curve values obtained from MRMCROC method for three radiologists in predicting lung nodule calcification on conventional gray scale radiography (GSR) and dual-energy radiography with color-representation (DCR)

Observer	Gray scale radiography	Dual-energy radiography with color-representation
1	0.871	0.949
2	0.739	0.957
3	0.874	0.926
Average	0.828	0.944

The difference in area under the receiver operating characteristic curve values between GSR and DCR imaging was statistically significant (*p* = 0.044)

*MRMCROC* multi-reader, multi-case receiver operating characteristic

## Discussion

Conventional chest radiography is a relatively low-cost, widely available modality performed with low radiation dose and remains mainstay of daily practice of chest radiology. However, the low sensitivity for small pulmonary nodule limits its application in detection of early lung cancer [3, 4]. With advances in digital chest radiography, development of new techniques including algorithms typically coupled with methodological innovations that use imaging physics to improve conspicuity, and image subtraction strategies has been shown to improve image quality of digital imaging systems over conventional film techniques [18, 19]. Dual-energy subtraction radiography is considered as one of emerging technologies that can distinguish bone from soft tissue by selective decomposition and visualization of bone and soft tissue images using the information about energy-dependent changes in the attenuation of different materials [20, 21]. Multiple studies have shown that dual-energy subtraction images allowed for improved detection of pulmonary nodules and differentiation of calcified and non-calcified lesions [6–12]. However, the value of the dual-energy subtraction technique remains debated in the literature, as different results have been published [22–24]. Furthermore one serious practical limitation of dual-energy subtraction radiography is that dual-energy subtraction radiography produces multiple images which need more burden for reading compared to single image from CR.

We hypothesized that dual-energy radiography with color-representation (DCR) may be useful for characterization of pulmonary nodules by expressing the degree of material density in terms of color intensity while nodule detection might be hampered by ghost artifact resulting from misregistration between the low- and high-energy images. This study evaluated the performance of DCR image using JAFROC and MRMCROC analysis and involved three observers and 65 cases with pairs of GSR and DCR from each patient (total 130 images). Our study showed that DCR image was indeed capable of improving the prediction of nodule calcification compared with GSR. And the performance of observers for nodule detection showed no statistically significant difference between two modalities.

Because color map provides enhanced contrast than conventional gray scale, research on the diagnostic utility of color radiography has been reported since the 1950’s [25–27]. In 1951, Donovan produced color radiographs by superimposing three exposures made with three different voltage X-ray beams. Each exposure was viewed through one of three color filters and full-color radiography was obtained [27]. These simulated color radiographs used color for the entire image, and achieved increased contrast for detail by sacrificing overall contrast [28, 29]. However, the techniques had not been widely accepted to date due to complicated processing methods and needs for skilled photographers using a projector or photographic paper [30].

Ogata et al. had evaluated the usefulness of color digital summation radiography [31, 32]. They proposed radiographs combined by additive color mixture method which comprised of two steps (the first step involved coloring previous and current radiographs, the second step involved summation of radiographs using the additive color mixture method), and displayed only areas of temporal change between previous and current radiographs in color. They evaluated 30 controls and 30 patients with newly detected solitary pulmonary nodules and six radiologists and five residents evaluated three image sets (set A, current and prior gray scale radiographs only ; set A with temporal subtraction images; set A with color digital summation radiography). There was no significant difference in ROC performance of six radiologists between three image sets. However, the residents showed significantly improved performance with temporal subtraction images and color digital summation radiography. And the mean reading time per case of all readers for color digital summation radiography was significantly shorter compared to gray scale radiography.

DCR in our study is, however, considerably different from color digital summation radiography in terms of obtaining and processing the image, and meaning of color hue, i.e. different color in DCR indicates different attenuating component, not temporal change. In detecting calcification of pulmonary nodules, DCR showed significantly increased performance as compared to GSR in three observers in our study (AUC GSR = 0.828, AUC DCR = 0.944; *p* < 0.04). Dual energy subtraction radiography also has been shown to be superior to conventional radiography in detecting calcification many studies [24, 33], except in a multicenter trial study reported by Ruhl et al. [22].

There was no published data available to date which compared dual energy subtraction image and color-represented image. Because DCR was derived from not by a subtraction method but by a color-adding compound method, it could display all the anatomic and density information at the same time. We believe that DCR has several advantages over the dual energy subtraction radiography: (1) Anatomic spatial relations between abnormalities and normal structure are readily appreciated. (2) DCR requires relatively small data and network storage because it can express additional information within a single image, while the dual energy subtraction radiography is composed of three or four images to display the whole information. (3) For the same reason, the reading time of radiologist with DCR may be shorter than with dual energy subtraction radiography. Some disadvantages of DCR could be overlapping anatomic clutter that cannot be eliminated and possible incongruity resulting from incorrect color mapping.

In our study, DCR was performed on a flat panel detector system with automatic exposure control determined tube load. The average entrance surface dose for DCR was 0.11 ± 0.04 mGy. It is less than 0.15 mGy for chest PA acquisition, recommended national reference dose on adult patients in the National Diagnostic Reference Levels (NDRLs) for the UK in 2010 [34].

There are several limitations in our study. First, we studied relatively small numbers of cases available for retrospective selected patient group and this might have limited the ability to distinguish existing differences between DCR and GSR imaging. Furthermore, although all cases were derived from clinical work-up, the frequency of disease in the set of testing radiographs is substantially higher than that would be seen in clinical practice. Third, since observations are done in a testing environment, it is less likely that observers missed nodules due to inattention, which may be an important factor in clinical practice. Finally, observers had no experience with DCR. Training might be needed to learn about the strengths and weaknesses of DCR.

## Conclusions

In summary, we compared the capability of lung nodule detection and characterization between DCR and conventional GSR. Even though DCR showed comparable performance in detecting a nodule when compared with conventional GSR, it provided superior performance in predicting presence of nodule calcification. Further investigations are forthcoming to analyze the clinical benefit of DCR and to establish its role in the daily routine exams.

## Abbreviations

AUC: Areas under curve
CR: Conventional radiography
CT: Computed tomography
DBM: Dorfman-Berbaum-Metz
DCR: Dual-energy radiography with color-representation
DER: Dual-energy radiography
FOM: Figure of merit
GSR: Gray scale chest radiography
JAFROC: Jackknife alternative free-response receiver operating characteristic
MRMCROC: Multi-reader, multi-case receiver operating characteristic
